# Interferon-Induced Transmembrane Proteins Inhibit Infection by the Kaposi’s Sarcoma-Associated Herpesvirus and the Related Rhesus Monkey Rhadinovirus in a Cell-Specific Manner

**DOI:** 10.1128/mBio.02113-21

**Published:** 2021-12-21

**Authors:** Bojan F. Hörnich, Anna K. Großkopf, Candice J. Dcosta, Sarah Schlagowski, Alexander S. Hahn

**Affiliations:** a Junior Research Group Herpesviruses, German Primate Center—Leibniz Institute for Primate Research, Göttingen, Germany; Icahn School of Medicine at Mount Sinai

**Keywords:** IFITMs, Kaposi's sarcoma-associated herpesvirus, influenza, interferons, virus entry

## Abstract

The interferon-induced transmembrane proteins (IFITMs) are broad-spectrum antiviral proteins that inhibit the entry of enveloped viruses. We analyzed the effect of IFITMs on the gamma-2 herpesviruses Kaposi’s sarcoma-associated herpesvirus (KSHV) and the closely related rhesus monkey rhadinovirus (RRV). We used CRISPR/Cas9-mediated gene knockout to generate A549 cells, human foreskin fibroblasts (HFF), and human umbilical vein endothelial cells (HUVEC) with combined IFITM1/2/3 knockout and identified IFITMs as cell-dependent inhibitors of KSHV and RRV infection in A549 cells and HFF but not HUVEC. IFITM overexpression revealed IFITM1 as the relevant IFITM that inhibits KSHV and RRV infection. Fluorescent KSHV particles did not pronouncedly colocalize with IFITM-positive compartments. However, we found that KSHV and RRV glycoprotein-mediated cell-cell fusion is enhanced upon IFITM1/2/3 knockout. Taken together, we identified IFITM1 as a cell-dependent restriction factor of KSHV and RRV that acts at the level of membrane fusion. Of note, our results indicate that recombinant IFITM overexpression may lead to results that are not representative for the situation at endogenous levels. Strikingly, we observed that the endotheliotropic KSHV circumvents IFITM-mediated restriction in HUVEC despite high IFITM expression, while influenza A virus (IAV) glycoprotein-driven entry into HUVEC is potently restricted by IFITMs even in the absence of interferon. Mechanistically, we found that KSHV colocalizes less with IFITM1 and IFITM2 in HUVEC than in A549 cells immediately after attachment, potentially contributing to the observed difference in restriction.

## INTRODUCTION

The family of interferon-induced transmembrane proteins (IFITMs) are small membrane proteins that exhibit antiviral activity toward a broad variety of viruses (1–6). There are five IFITMs present in the human genome, but only IFITM1, IFITM2, and IFITM3 are known to be immune related and interferon (IFN) inducible (reviewed in references 6 and 7). IFITM1 localizes to the plasma membrane, while IFITM2 and IFITM3 localize to endosomes/lysosomes (5, 8).

The exact mechanism of IFITM-mediated restriction of viral replication is not completely understood. It is, however, clear that restriction occurs mainly at the viral entry stage (3, 9, 10). According to some reports, IFITMs modify the overall membrane fusogenicity by modification of the membrane lipid composition and/or the membrane rigidity and thus prevent virus-host membrane fusion (11–14), probably causing arrest of the fusion pore opening following hemifusion (11, 12, 15). Other modes of action, such as, e.g., recruitment of additional antiviral factors, altered endocytic trafficking, and interference with vacuolar ATPase, have been postulated as well (reviewed in reference 16).

The majority of IFITM-restricted viruses are RNA viruses. The interplay of IFITMs with DNA viruses has been studied less extensively and with more ambiguous results. While vaccinia virus and herpes simplex virus 1 (HSV-1) are restricted by overexpression of individual IFITM proteins (17, 18), human papillomavirus 16 (HPV16) and the nonenveloped adenovirus type 5 are not (19). Interestingly, for the human cytomegalovirus (HCMV), small interfering RNA (siRNA)-mediated IFITM knockdown resulted in reduced infection and disturbed virus assembly (20). Varying results were obtained for Epstein-Barr virus (EBV), a gammaherpesvirus. While the initial entry of EBV was enhanced by overexpression of IFITM1 (21, 22), incorporation of IFITM2/3 into viral particles reduced the infectivity of progeny virus, whereas IFITM1 incorporation had no effect (23). Together, the literature on IFITM-mediated effects on the alphaherpesvirus HSV-1, the betaherpesvirus HCMV, and the gammaherpesvirus EBV indicate differences in the activity of IFITM proteins toward different herpesvirus subfamilies.

The Kaposi’s sarcoma-associated herpesvirus (KSHV) and the related rhesus monkey rhadinovirus (RRV) belong to the gammaherpesvirus subfamily (24). KSHV is associated with Kaposi’s sarcoma (KS), multicentric Castleman’s disease, primary effusion lymphoma (reviewed in reference 25), osteosarcoma (26), and KSHV inflammatory cytokine syndrome (KICS) (27). The incidence of KSHV-related disease and KSHV seroprevalence are low in industrial countries (28, 29), but KSHV represents a significant health burden in sub-Saharan Africa, where KSHV-related cancers are common (30, 31).

KSHV and RRV exhibit broad cell tropism *in vitro* (32, 33). Both viruses encode a set of glycoproteins (g) that mediate entry and are conserved among herpesviruses. Of these, gH, gL, and gB are the most extensively studied (reviewed in reference 34). KSHV and RRV enter many cell types through the interaction of the gH/gL complex with members of the ephrin receptor tyrosine kinase family (Ephs) (35–37) and, in the case of RRV, also with members of the plexin domain-containing protein family (38). KSHV also interacts with heparan sulfate and integrins (39–41). Entry of both viruses occurs mainly via endocytotic routes (33, 42–44). Following internalization, the viral membrane fuses with the host membrane. Several reports implicate the gH/gL complex together with gB as the minimal set of glycoproteins required for membrane fusion (38, 45, 46).

One study reported an enhancing role of IFITMs in the infection of the BJAB B cell line and human dermal microvascular endothelial cells (HMVEC-D) cells by KSHV, EBV, and herpes simplex virus 2 (HSV-2) (21). However, given the considerable differences between KSHV and RRV entry into B cells and different adherent cells (33, 36, 37), in particular since KSHV infection of B cell lines is, with a few exceptions, efficient only through cell-to-cell transfer (47–49), we hypothesized that IFITM-mediated restriction may be dependent on the nature of the target cell. Another question that we sought to address is whether IFITMs restrict RRV in human cells.

## RESULTS

### KSHV induces IFITM expression in A549 cells.

**FIG 1:**
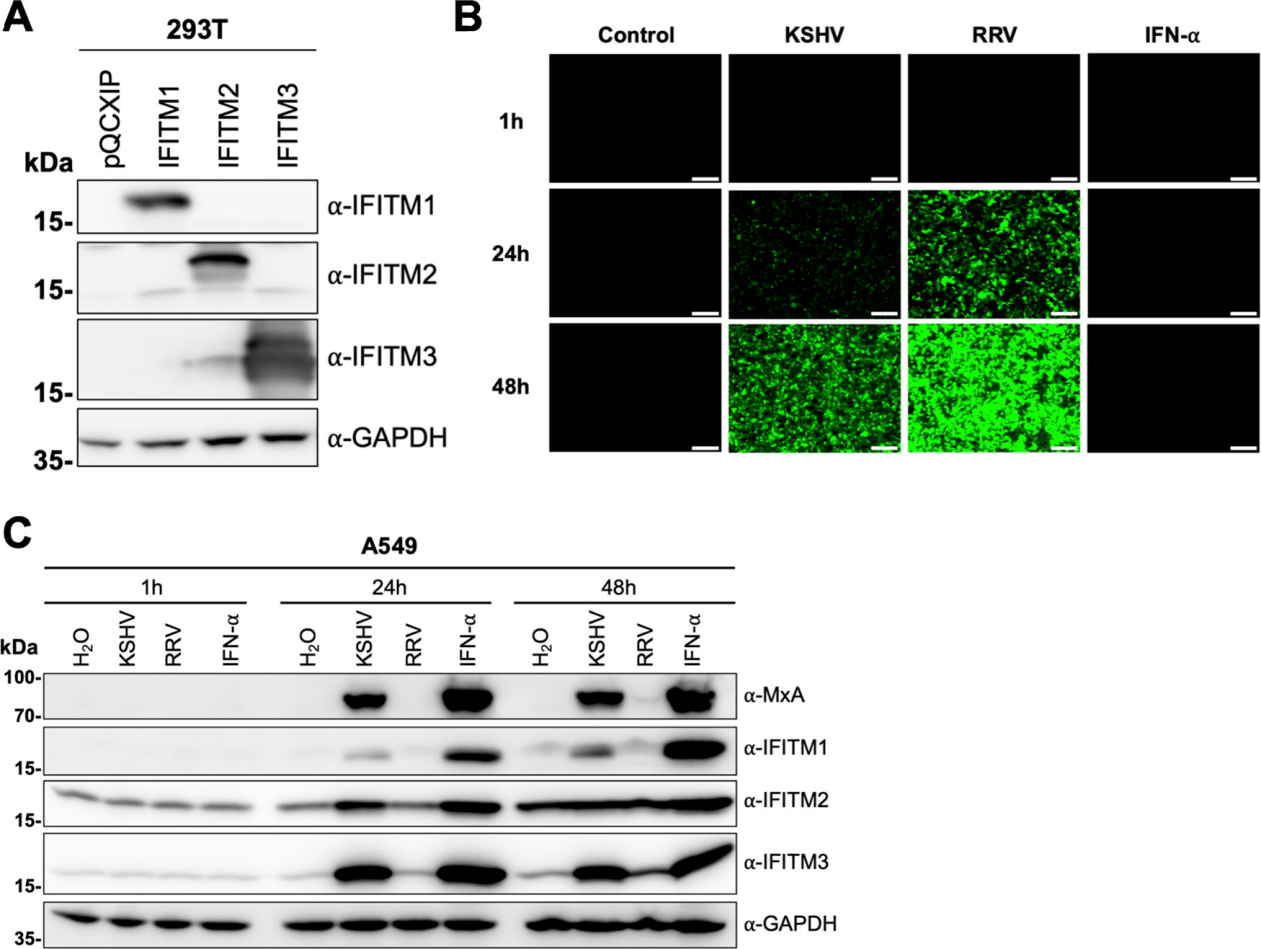
KSHV induces IFITM1, IFITM2, and IFITM3 expression in A549 cells. (A) Western blot of 293T cells transduced with pQCXIP constructs to express IFITM1 to -3 or pQCXIP (empty vector). IFITMs were detected using the respective IFITM antibody, and GAPDH served as a loading control. (B and C) Fluorescence microscopy images (scale bar, 200 μm) (B) and Western blot analysis (C) of A549 cells infected with KSHV-GFP or RRV-YFP or treated with H_2_O or IFN-α (5,000 U/ml) for the indicated time and harvested using SDS sample buffer. IFITM expression was detected with antibodies shown in panel A. MxA served as control for IFN-stimulated gene induction; GAPDH served as a loading control.

We first validated the specificity of the antibodies used in this study for Western blot analysis after directed expression (Fig. 1A). Next, we examined expression of IFITM proteins at baseline levels and after stimuli such as virus infection in the human lung epithelial cell line A549, which has been well characterized with regard to IFN signaling and IFITM expression (50–53). We infected the cells with KSHV BAC16 recombinant virus carrying a green fluorescent protein (GFP) reporter gene and RRV-YFP carrying a yellow fluorescent protein (YFP) reporter gene (Fig. 1B). Treatment with H_2_O and IFN-α served as negative and positive controls for IFITM induction, respectively. IFITM2 and IFITM3 were detected at low levels without IFN treatment, while IFITM1 and human myxovirus resistance protein 1 (MxA), another IFN-induced protein, were not detectable without stimulation (Fig. 1C). At the 1-h time point, neither treatment induced IFITM or MxA expression relative to the background. At the 24-h time point, induction over background levels of IFITM1, IFITM2, IFITM3, and MxA was observed in IFN-α-treated or KSHV-infected cells but not in RRV-infected cells. At 48 h, IFITM3 was also slightly induced by RRV, and IFITM2 induction relative to H_2_O treatment was barely discernible anymore. Basal IFITM expression also increased slightly over time after plating. In summary, KSHV-containing inoculum and IFN-α induced IFITM expression.

### Triple knockout of IFITM1/2/3 enhances KSHV and RRV infection of A549 cells and human foreskin fibroblasts (HFF).

**FIG 2:**
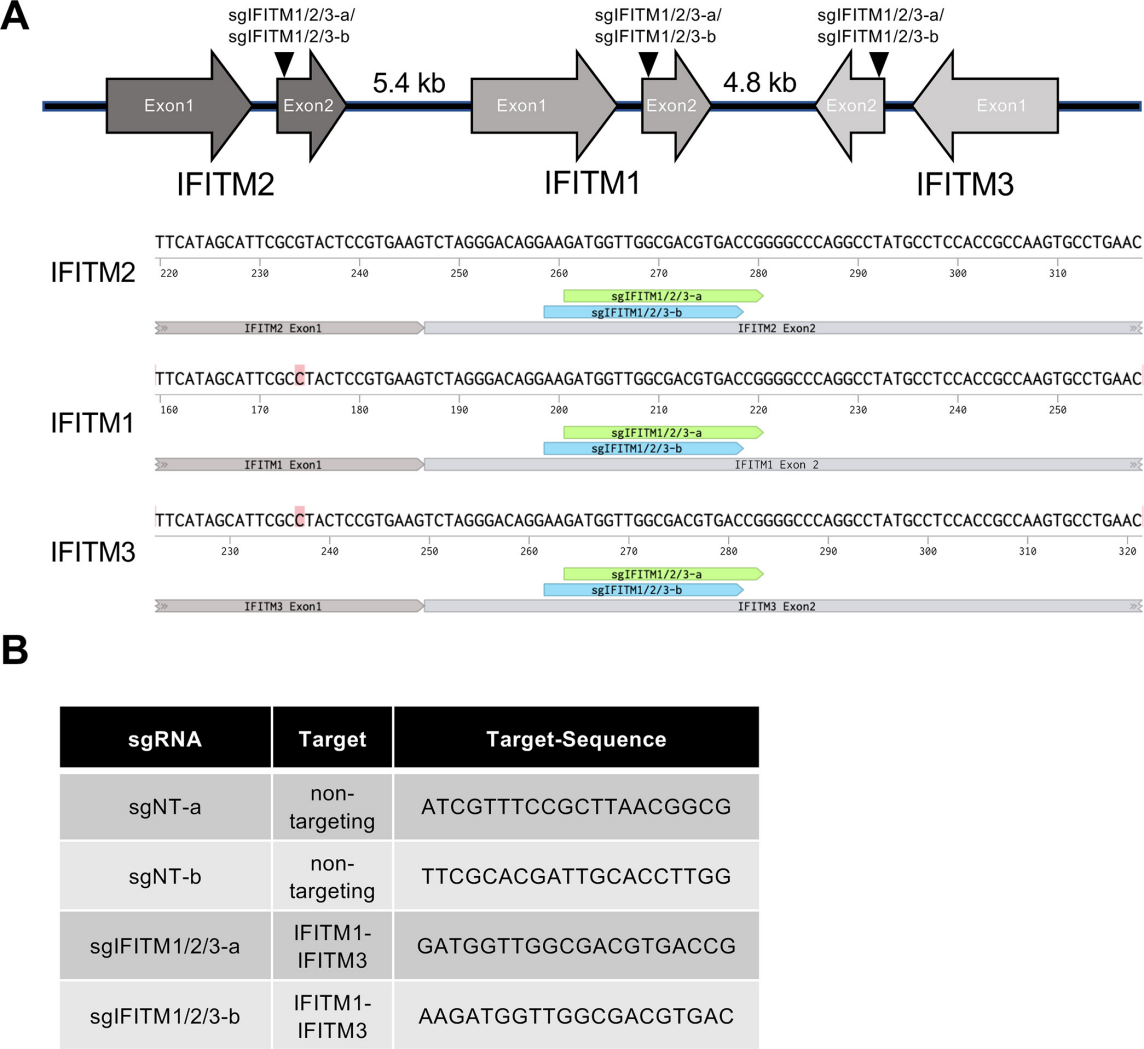
Localization of the IFITM cluster on chromosome 11 in the human genome and sgRNAs used in this study. (A) Upper panel, schematic drawing (not to scale) of the localization of IFITM1, IFITM2, and IFITM3 on chromosome 11 in the human genome with target sites of sgRNAs targeting exon2 of IFITM1 to -3 (sgIFITM1/2/3-a, sgIFITM1/2/3-b). Lower panel, alignment of the target sites of sgIFITM1/2/3-a and sgIFITM1/2/3-b. (B) Sequences of sgRNAs used in this study.

Overexpression of IFITMs alters their subcellular localization (6; our observations), IFITMs are usually induced together, and recent studies report that IFITMs form homo- and hetero-oligomers (54–56) and might thus act synergistically. We therefore used CRISPR/Cas9 to generate triple IFITM1/2/3 knockout cells to study the effects of basal IFITM expression as well as IFN-induced IFITM expression on KSHV and RRV infection. We identified two single guide RNAs (sgRNAs) (sgIFITM1/2/3-a, sgIFITM1/2/3-b), which target the second exon of all three immune-related IFITMs (Fig. 2A and B). These sgRNAs were transduced together with Cas9 using the lentiCRISPRv2 system (57).

**FIG 3:**
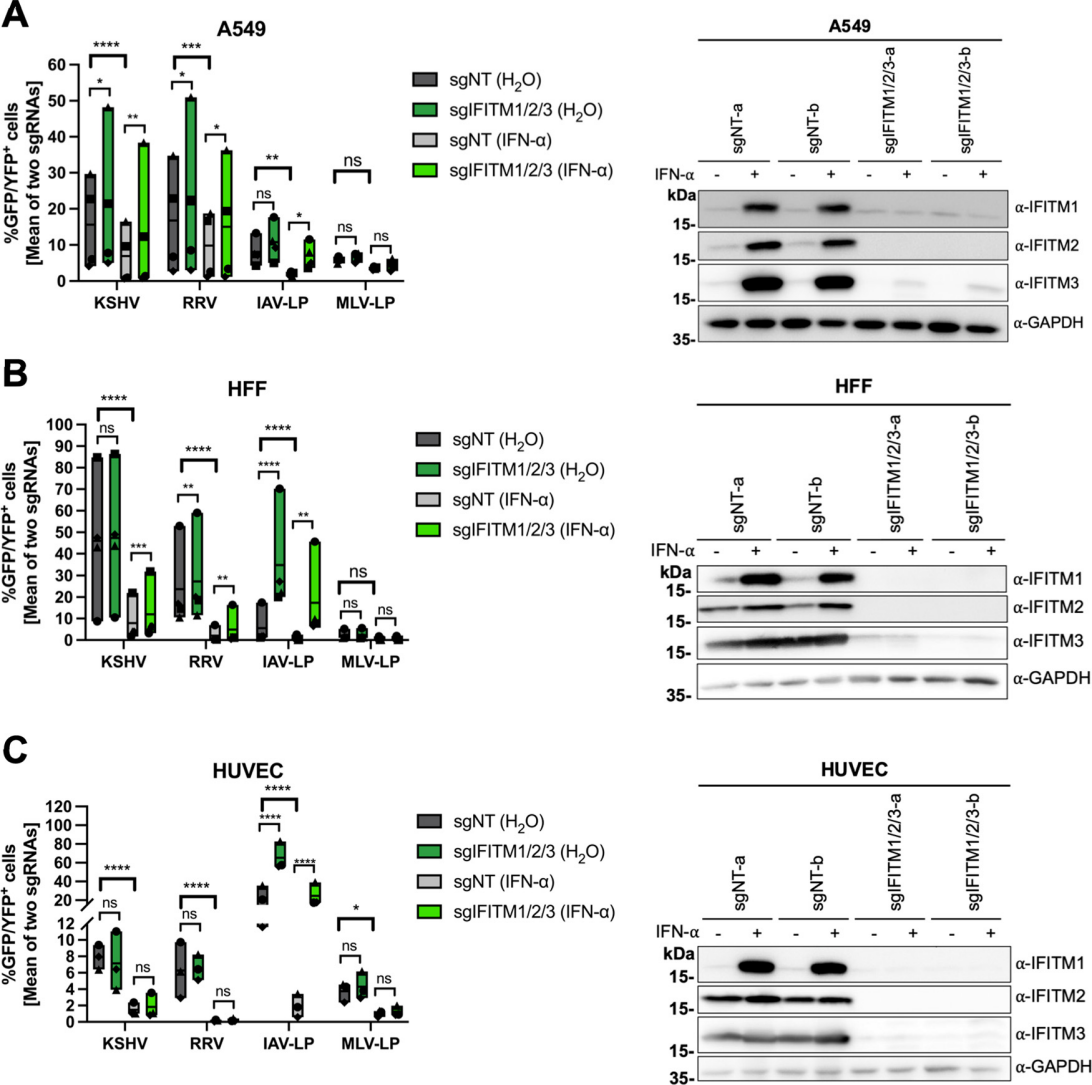
IFITM1/2/3 triple knockout enhances KSHV and RRV infection in A549 cells and HFF. A549 cells (A), HFF (B), and HUVEC (C) were transduced with lentiviral vectors encoding Cas9 and the sgRNAs shown in Fig. 2. (A to C, left panels) IFITM knockout (sgIFITM1/2/3-a, sgIFITM1/2/3-b) or control (sgNT-a, sgNT-b) cells treated with IFN-α (5,000 U/ml) or H_2_O (control) and infected with KSHV-GFP, RRV-YFP, IAV lentiviral pseudotype (IAV-LP), or MLV lentiviral pseudotype (MLV-LP). Infection was measured using flow cytometry to detect expression of the fluorescent reporter gene. The graph shows individual data points representing averaged values for GFP^+^/YFP^+^ cells of either two nontargeting (sgNT-a, sgNT-b) or IFITM1/2/3 knockout (sgIFITM1/2/3-a, sgIFITM1/2/3-b) transduced cells and floating bars representing the mean averaged from results of four independent experiments for A549 cells and HFF (A and B) and three independent experiments for HUVEC (C). Infections for each single experiment were performed in triplicate for each condition. Data points from the same experiment are labeled with identical symbols. The different sgRNAs were treated as biological replicates within each experiment. Statistical significance was determined by two‐way analysis of variance (ANOVA), and *P* values were corrected for all possible multiple comparisons within one family by Tukey’s method (nonsignificant [ns], *P* > 0.05; *, *P* ≤ 0.05; **, *P* ≤ 0.01; ***, *P* ≤ 0.001; ****, *P* ≤ 0.0001). (A to C, right panels) Representative Western blots of IFITM knockout (sgIFITM1/2/3-a or sgIFITM1/2/3-b) or control (sgNT-a or sgNT-b) cells treated with IFN-α (5,000 U/ml) or H_2_O. Indicated IFITM expression was detected with antibodies shown in Fig. 1A; GAPDH served as a loading control.

We chose the lung epithelial cell line A549 as an epithelial cell model. KSHV is occasionally detected in lung tissue (58), and A549 cells are well characterized with regard to IFITM-mediated restriction of different viruses (1, 9, 53). HFF were chosen as a fibroblast model and human umbilical vein endothelial cells (HUVEC) as a model for endothelial cells. Knockout or substantial knockdown of IFITM1, IFITM2, and IFITM3 was achieved (Fig. 3A to C, right panels). Lentiviral particles (LP) encoding a GFP reporter gene pseudotyped with influenza A virus (IAV)-hemagglutinin (HA)/neuraminidase (NA) (IAV-LP) served as a positive control for IFITM-mediated restriction, while particles pseudotyped with IFITM-resistant amphotropic murine leukemia virus (MLV) envelope (MLV-LP) served as a negative control (1). Infections were performed with or without prior IFN-α stimulation. IFN-α treatment resulted in a significant reduction of KSHV, RRV, and IAV-LP infection in A549 cells (Fig. 3A, left panel). Both KSHV and RRV infection were enhanced in non-IFN-α-treated IFITM1/2/3 knockout A549 cells, indicating that basal IFITM levels or IFITM expression induced upon contact with the inoculum affect KSHV and RRV infection of A549 cells. In IFN-α-treated IFITM1/2/3 knockout cells, infection nearly reached levels of control-treated sgNT-transduced cells. IAV-LP infection was increased upon IFITM1/2/3 knockout, while MLV-LP infection was not affected by IFITM1/2/3 knockout in A549 cells, in keeping with published results (1).

IFN-α pretreatment reduced KSHV and RRV infection of HFF more potently than infection of A549 cells (Fig. 3B, left panel). However, IFITM1/2/3 knockout in HFF enhanced KSHV infection only of IFN-α-treated cells, while RRV infection was slightly but significantly enhanced in both IFN-α and control-treated IFITM1/2/3 knockout cells. We observed relatively high basal IFITM2/3 expression in HFF, which was only marginally increased by IFN-α (Fig. 3B, right panel). Infection of IFN-α-treated IFITM1/2/3 knockout HFF by KSHV or RRV did not reach levels of untreated sgNT-transduced cells, unlike what was observed with A549 cells, suggesting that IFITM-mediated restriction of KSHV and RRV infection plays a comparatively minor role in the overall IFN-α-mediated restriction of these two herpesviruses in HFF. The most potent effect of IFITM1/2/3 knockout was observed with IAV-LP infection, which was increased in both IFN-α- and control-treated cells. MLV-LP infection of HFF was not significantly affected by IFITM1/2/3 knockout.

Like HFF, HUVEC expressed IFITM2 and IFITM3 at high basal levels (Fig. 3C, right panel). IFN-α treatment of HUVEC resulted in a reduction of KSHV infection and an even more pronounced reduction of RRV infection (Fig. 3C, left panel). However, IFITM1/2/3 knockout had no significant effect on KSHV or RRV infection. Again, IAV-LP infection was strongly enhanced by IFITM1/2/3 knockout in both IFN-α- and control-treated HUVEC, while MLV-LP was not affected.

Overall, these results demonstrate IFITM-mediated restriction of KSHV and RRV infection of A549 cells and HFF but not HUVEC.

### IFITM1 overexpression reduces KSHV and RRV infection in a cell-dependent manner.

**FIG 4:**
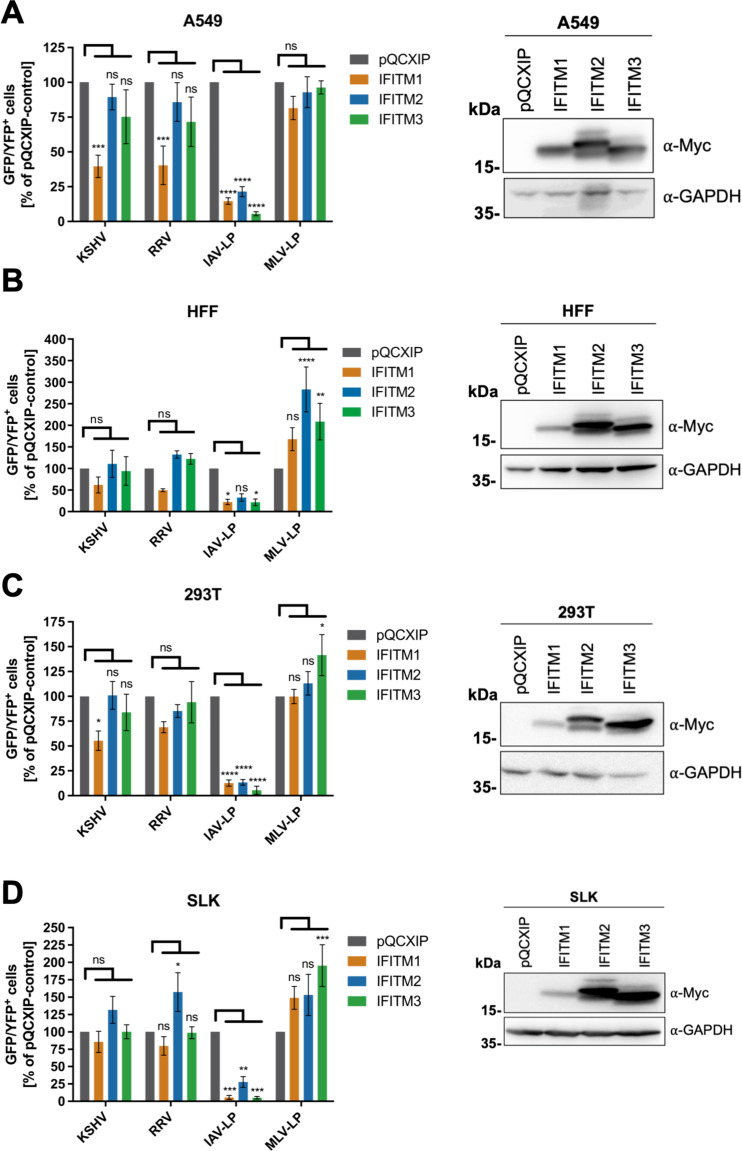
Overexpression of IFITM1 inhibits KSHV and RRV infection in a cell-specific manner. A549 cells (A), HFF (B), 293T cells (C), and SLK cells (D) were transduced with pQCXIP constructs to express IFITM1-3 or pQCXIP (empty vector). (A to D, left panels) IFITM-overexpressing cells were infected with KSHV-GFP, RRV-YFP, IAV lentiviral pseudotype (IAV-LP), or MLV lentiviral pseudotype (MLV-LP). Infection was measured using flow cytometry to detect expression of the fluorescent reporter genes. The data show values normalized to pQCXIP empty vector, which was set to 100%, and the error bars represent the standard error of the mean of results of four independent experiments, each performed in triplicate. Statistical significance was determined by ordinary two-way ANOVA, and *P* values were corrected for multiple comparisons by Dunnett’s method (ns, *P* > 0.05; *, *P* ≤ 0.05; **, *P* ≤ 0.01; ***, *P* ≤ 0.001; ****, *P* ≤ 0.0001). (A to C, right panels) Representative Western blots of IFITM-overexpressing cells. Expression of myc-tagged IFITMs was determined using anti-myc antibody; GAPDH served as a loading control.

We next investigated the effect of individual IFITMs through directed expression by retroviral transduction (Fig. 4A to D) and included the following additional cell lines: (i) 293T cells as another cell line of either epithelial or neuroendocrine origin (59) and (ii) SLK cells, a clear renal carcinoma cell line (60) that is an established model for KSHV infection and propagation (61).

Overexpression of IFITM1 in A549 reduced KSHV and RRV infection by over 50%, whereas overexpression of IFITM2 and IFITM3 resulted in only a nonsignificant reduction (Fig. 4A, left panel), identifying IFITM1 as the IFITM that restricts KSHV and RRV in A549 cells. In agreement with the results in IFITM1/2/3 knockout experiments and published results (1), IAV-LP infection was reduced by all IFITMs, most prominently by IFITM3, and infection of MLV-LP was not affected. As directed expression of IFITM3 led to a slight nonsignificant reduction in KSHV/RRV infection, we tested whether this effect would change through the introduction of well-characterized mutations into IFITM3 that change its subcellular localization from predominantly endosomal to a broader distribution (62, 63). While in this set of experiments in A549 cells (see Fig. S1A), effects were of similar magnitude as before, the ∼30% reduction of KSHV infection by IFITM3 reached significance. Interestingly, this mild effect on KSHV infection was reduced by deletion of amino acids 1 to 21 (Δ1–21) or by Y20A mutation in the N-terminal domain (62) or by the 43AS mutation (amino acids 43 to 48 changed to alanines) in the conserved intracellular loop (63). RRV infection, in contrast, was inhibited by IFITM3 bearing the mutations Δ1–21 or Y20A but not by IFITM3 wild type (wt) or the 43AS mutant. Taken together, these results clearly implicate IFITM3’s subcellular sorting motifs as major determinants of activity in particular against RRV.

10.1128/mBio.02113-21.1 FIG S1 Overexpression of IFITM localization mutants and IFITM1 to -3 in HUVEC. (A and B) A549 cells were transduced with pQCXIP constructs to express pQCXIP (empty vector), IFITM1, IFITM3, and IFITM3 43AS, Y20A, and Δ1–21 mutants (A), and HUVEC were transduced with pQCXIP constructs to express IFITM1 to -3 or pQCXIP (empty vector) (B). IFITMs overexpressing cells were infected with KSHV-GFP, RRV-YFP, IAV lentiviral pseudotype (IAV-LP), or MLV lentiviral pseudotype (MLV-LP). Infection was measured using flow cytometry to detect expression of the fluorescent reporter genes. The data show values normalized to pQCXIP empty vector, which was set to 100%, and the error bars represent the standard error of the mean of results of four independent experiments for A549 cells and standard deviation of results of three independent experiments for HUVEC, each performed in triplicate. Statistical significance was determined by ordinary two-way ANOVA; *P* values were corrected for multiple comparisons by Dunnett’s method (ns, *P* > 0.05; *, *P* ≤ 0.05; **, *P* ≤ 0.01; ***, *P* ≤ 0.001; ****, *P* ≤ 0.0001). (A and B, right panels) Representative Western blots of IFITM-overexpressing cells. Expression of myc-tagged IFITMs was determined using anti-myc antibody, and anti-IFITM3 antibody was used to detect untagged IFITM3 mutants; the Δ1–21 mutant is less well detected due to IFITM3 antibody epitope. GAPDH served as a loading control. (C) Bright-field microscopy images of HUVEC transduced with pQCXIP constructs to express IFITM1 to -3 or pQCXIP (empty vector) at different magnifications. Download FIG S1, TIF file, 2.0 MB. Copyright © 2021 Hörnich et al. 2021 Hörnich et al. https://creativecommons.org/licenses/by/4.0/ This content is distributed under the terms of the Creative Commons Attribution 4.0 International license.

IFITM1 overexpression also reduced RRV and KSHV infection of HFF (Fig. 4B, left panel), but the effect did not reach statistical significance, mostly because of a rather high pooled variance in this set of experiments, which may reflect the primary nature of HFF combined with comparatively high constitutive IFITM expression. In addition, we observed a nonsignificant but noticeable enhancement of RRV infection in IFITM2/3-overexpressing HFF. Again, these observations are in agreement with the effects observed in IFITM1/2/3 knockout HFF. While IAV-LP infection was reduced in HFF, MLV-LP infection was enhanced by overexpression of all IFITMs, significantly for IFITM2 and IFITM3.

A trend similar to that observed in A549 cells was also observed in 293T cells (Fig. 4C, left panel): IFITM1 overexpression reduced KSHV and RRV infection, although not significantly for RRV. MLV-LP infection was slightly increased by overexpression of IFITM3 in 293T cells.

In HUVEC, IFITM1 and IFITM3 overexpression also slightly decreased KSHV and RRV infection (Fig. S1B). Surprisingly, we also observed inhibition of MLV-LP by IFITM1 and IFITM2 in this setting. Further, we noticed that HUVEC transduced to overexpress IFITMs exhibited a highly abnormal morphology (Fig. S1C).

A different observation was made in SLK cells (Fig. 4D, left panel), where neither IFITM1 nor IFITM3 overexpression resulted in reduced KSHV or RRV infection. Again, IFITM2 overexpression in SLK cells slightly enhanced KSHV infection and significantly enhanced RRV infection. An enhancement of infection by all IFITMs was observed with MLV, significantly for IFITM3.

Taken together, our IFITM overexpression experiments corroborated the results observed in our IFITM1/2/3 knockout experiments and a cell-specific activity of individual IFITMs toward KSHV and RRV. Even if recombinant overexpression in HUVEC had some effect, this does not reflect the situation at endogenous expression levels. Restriction of the typically nonrestricted MLV-LP and drastically aberrant morphology with a “foamy” appearance (Fig. S1C) suggest that recombinantly overexpressed IFITMs in HUVEC, which naturally already express IFITM2 and IFITM3 at high levels, may lead to visibly distorted cellular membranes and vesicles and nonspecific effects on infection. Furthermore, IFITM1 was identified as the major contributor to IFITM-mediated restriction of KSHV and RRV.

### KSHV entry pathways and colocalization with IFITMs differ between A459 cells and HUVEC.

**FIG 5:**
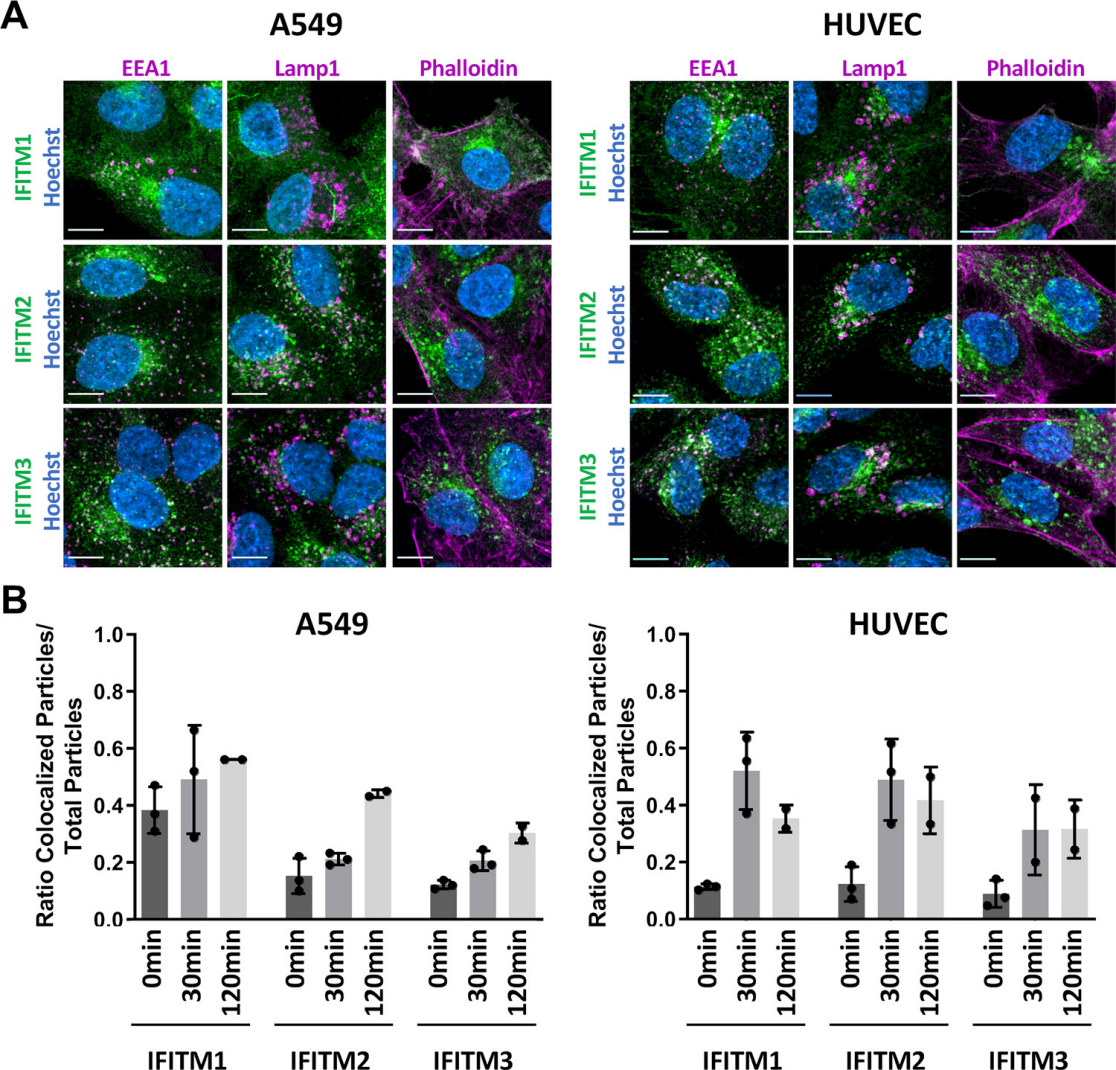
Kinetics of colocalization of KSHV and IFITMs differ between HUVEC and A549 cells. (A) Confocal microscopy images of A549 cells (left panel) and HUVEC (right panel) treated with IFN-α (5,000 U/ml) for 16 h and stained with IFITM1, IFITM2, or IFITM3 antibody (all green). Costaining was performed with antibodies to EEA1, LAMP1, or phalloidin conjugate (all magenta) and Hoechst stain (blue). Scale bars, 10 μm. (B) A549 cells (left panel) and HUVEC (right panel) were treated with IFN-α (5,000 U/ml) for 16 h and infected with KSHV_mNeon-orf65. Colocalization of IFITM and mNeon signals was quantified. Data are shown as individual biological replicates together with the mean, and error bars represent the standard deviation. Per biological replicate and time point, at least three images were analyzed.

IFITM localization was reported to play a critical role in their antiviral effect (64, 65). For the highly restricted IAV, IFITM3-mediated restriction might be partially explained by the observation that IAV specifically colocalizes with IFITM3-positive vesicles (15, 53). As expected, we observed different subcellular localizations of IFITM1, IFITM2, and IFITM3 in both IFN-α-treated A549 cells and HUVEC (Fig. 5A; Fig. S2). IFITM2 and IFITM3 partially colocalized with the early endosome marker EEA1 and with the endolysosomal marker LAMP1, while IFITM1 localized to the plasma membrane and was distributed more toward the perimeter of the cell. IFITM1 was also found colocalized with EEA1 and LAMP1, particularly in HUVEC, but did generally show a less pronounced vesicular localization than IFITM2/IFITM3. As we observed IFITM1-mediated inhibition of KSHV and RRV infection in A549 cells, we examined how these viruses enter A549 by probing different entry pathways with inhibitors (Fig. S3). IFITM1 was reported to restrict viruses that directly fuse at the plasma membrane (18). KSHV and RRV infection of A549 cells and HUVEC was sensitive to bafilomycin A1 (Fig. S3A), indicating dependence on vesicular acidification, and were sensitive to methyl-β-cyclodextrin (MBCD) (Fig. S3B), commonly believed to indicate a role for cholesterol-rich membrane domains (66, 67). 5-(*N*-Ethyl-*N*-isopropyl)amiloride (EIPA; an inhibitor of macropinocytosis [68]) had at best a marginal effect on KSHV and RRV on A549 cells, suggesting that neither KSHV nor RRV enter A549 cells predominantly via macropinocytosis. KSHV infection of HUVEC on the other hand was sensitive to EIPA (Fig. S3C), in agreement with published data (42) and suggesting macropinocytotic entry, whereas RRV infection of HUVEC was not EIPA sensitive. It should be noted that EIPA and MBCD at the first significantly effective concentrations caused minor yet significant changes in cellular ATP content (up to −20%) in our cell viability assay (Fig. S3D).

10.1128/mBio.02113-21.2 FIG S2 Localization of IFITM1/2/3 in A549 cells and HUVEC. (A and B) Confocal microscopy images of A549 cells (A) and HUVEC (B) treated with IFN-α (5,000 U/ml) for 16 h and stained with IFITM1, IFITM2, or IFITM3 antibody (all green). Costaining was performed with antibodies to EEA1, LAMP1, or phalloidin conjugate (all magenta) and Hoechst stain (blue). Scale bars, 10 μm. Download FIG S2, JPG file, 2.3 MB. Copyright © 2021 Hörnich et al. 2021 Hörnich et al. https://creativecommons.org/licenses/by/4.0/ This content is distributed under the terms of the Creative Commons Attribution 4.0 International license.

10.1128/mBio.02113-21.3 FIG S3 KSHV enters A549 cells via an endosomal pH-dependent, macropinocytosis-independent pathway. (A to C) A549 cells (left panel) and HUVEC (right panel) were pretreated for 30 min with twice the indicated concentration of either bafilomycin A1 (A), MBCD (B), or EIPA (C) and incubated with KSHV-GFP, RRV-YFP, IAV lentiviral pseudotype (IAV-LP), or MLV lentiviral pseudotype (MLV-LP) to the final concentration. In the case of EIPA treatment, the medium was changed after 12 h due to cytotoxicity of prolonged incubation. Infection was measured after 48 h using flow cytometry to detect expression of the fluorescent reporter genes. The data show values normalized to the respective solvent control, which was set to 100%, and the error bars represent the standard deviation of the results of one representative experiment performed in triplicate (in duplicate for KSHV with EIPA). Statistical significance was determined by ordinary two-way ANOVA; *P* values were corrected for multiple comparisons by Dunnett’s method (ns, *P* > 0.05; *, *P* ≤ 0.05; **, *P* ≤ 0.01; ***, *P* ≤ 0.001; ****, *P* ≤ 0.0001). (D) Cytotoxicity of substances was determined via a CellTiter-Glo assay. A549 cells (left panel) and HUVEC (right panel) were incubated with the substances at the indicated concentrations. In the case of EIPA treatment, the medium was changed after 12 h due to cytotoxicity of prolonged incubation. After 48 h, cell viability was determined using the luciferase-based CellTiter-Glo assay. The data show values normalized to the respective solvent control, which was set to 100%, and the error bars represent the standard deviation of the results of one representative experiment performed in triplicate. Statistical significance was determined by ordinary one-way ANOVA; *P* values were corrected for multiple comparisons by Dunnett’s method (ns, *P* > 0.05; *, *P* ≤ 0.05; **, *P* ≤ 0.01; ***, *P* ≤ 0.001; ****, *P* ≤ 0.0001). Download FIG S3, TIF file, 1.6 MB. Copyright © 2021 Hörnich et al. 2021 Hörnich et al. https://creativecommons.org/licenses/by/4.0/ This content is distributed under the terms of the Creative Commons Attribution 4.0 International license.

We next analyzed colocalization of KSHV particles with IFITMs in A549 cells and HUVEC. We utilized a KSHV_mNeon-orf65, which is tagged with mNeonGreen at the capsid protein orf65, to visualize virions in IFN-α-treated cells at different time points (Fig. 5B; Fig. S4). KSHV_mNeon-orf65 particles were detectable at the perimeter at the 0-min time point and were detected inside the cells from the 30-min time point on. Some particles reached the nucleus at the 120-min time point. As IFITMs are widely distributed throughout the cell, partial overlap with KSHV_mNeon-orf65 particles was observed for all IFITMs, most prominently at later time points. While colocalization with all IFITMs followed roughly the same pattern over time in HUVEC, A549 cells exhibited differences between IFITM1 and IFITM2/3. In A549 cells, KSHV capsids were found to colocalize to the same degree with IFTIM1 at all time points, while HUVEC showed a considerably lower extent of mNEON-IFITM1 overlap at the 0-min time point. While some particles localized to areas of high intensity in the IFITM staining, KSHV_mNeon-orf65 particles were also frequently found in regions with overall lower IFITM signal. These areas were often adjacent to IFITM-positive areas, which might be compatible with the luminal spaces of large vesicles.

10.1128/mBio.02113-21.4 FIG S4 KSHV virus particles in A549 cells and HUVEC. Representative confocal microscopy images of A549 cells (A) and HUVEC (B) treated with 5,000 U/ml IFN-α, infected with KSHV_mNeon-orf65 (green), and used for the quantification shown in Fig. 5B. Staining was performed using IFITM1, IFITM2, or IFITM3 antibody (magenta) and Hoechst stain (blue). Scale bars, 10 μm. Download FIG S4, JPG file, 2.8 MB. Copyright © 2021 Hörnich et al. 2021 Hörnich et al. https://creativecommons.org/licenses/by/4.0/ This content is distributed under the terms of the Creative Commons Attribution 4.0 International license.

### KSHV and RRV glycoprotein-mediated cell-cell fusion is reduced by IFITMs.

IFITMs were reported to modulate overall membrane fusogenicity and thereby entry of viral particles (11, 12, 69). We therefore utilized a cell-cell fusion assay to determine whether KSHV and RRV glycoprotein-mediated fusion activity is modulated by IFITMs. 293T effector cells were transfected with KSHV gH/gL or RRV gH/gL together with RRV gB and a plasmid encoding a VP16-Gal4 transactivator fusion protein. RRV gB was used because KSHV gB does not allow for efficient cell-cell fusion (46). Because of the low fusion activity of RRV gH/gL+gB, cell-cell fusion was analyzed after 48 h to reach robust levels of fusion activity (Fig. S5). Transfected effector cells were added to IFN-α-treated A549 IFITM1/2/3 knockout cells transduced with a lentiviral Gal4-driven TurboGFP-luciferase reporter construct or 293T cells transfected to express IFITM1, IFITM2, or IFITM3 and a Gal4-driven TurboGFP-luciferase reporter construct. Luciferase activity was measured as a readout for fusion.

10.1128/mBio.02113-21.5 FIG S5 Cell‐cell fusion assay. Effector cells (293T cells transfected with either empty vector [eV] or expression plasmids for the indicated viral glycoproteins together with Vp16‐Gal4 expression plasmid) were added to target cells (A549 cells transduced with a Gal4-driven TurboGFP-luciferase construct). After the indicated time points, luciferase activity was measured. The data show fold values relative to empty vector control effector cells. Error bars represent the standard deviation of the results of one representative experiment performed in triplicate. Statistical significance was determined by two‐way ANOVA; *P* values were corrected for multiple comparisons by Dunnett’s method (ns, *P* > 0.05; **, *P* ≤ 0.05; ***, *P* ≤ 0.01; ***, *P* ≤ 0.001; ****, *P* ≤ 0.0001). Download FIG S5, TIF file, 0.5 MB. Copyright © 2021 Hörnich et al. 2021 Hörnich et al. https://creativecommons.org/licenses/by/4.0/ This content is distributed under the terms of the Creative Commons Attribution 4.0 International license.

**FIG 6:**
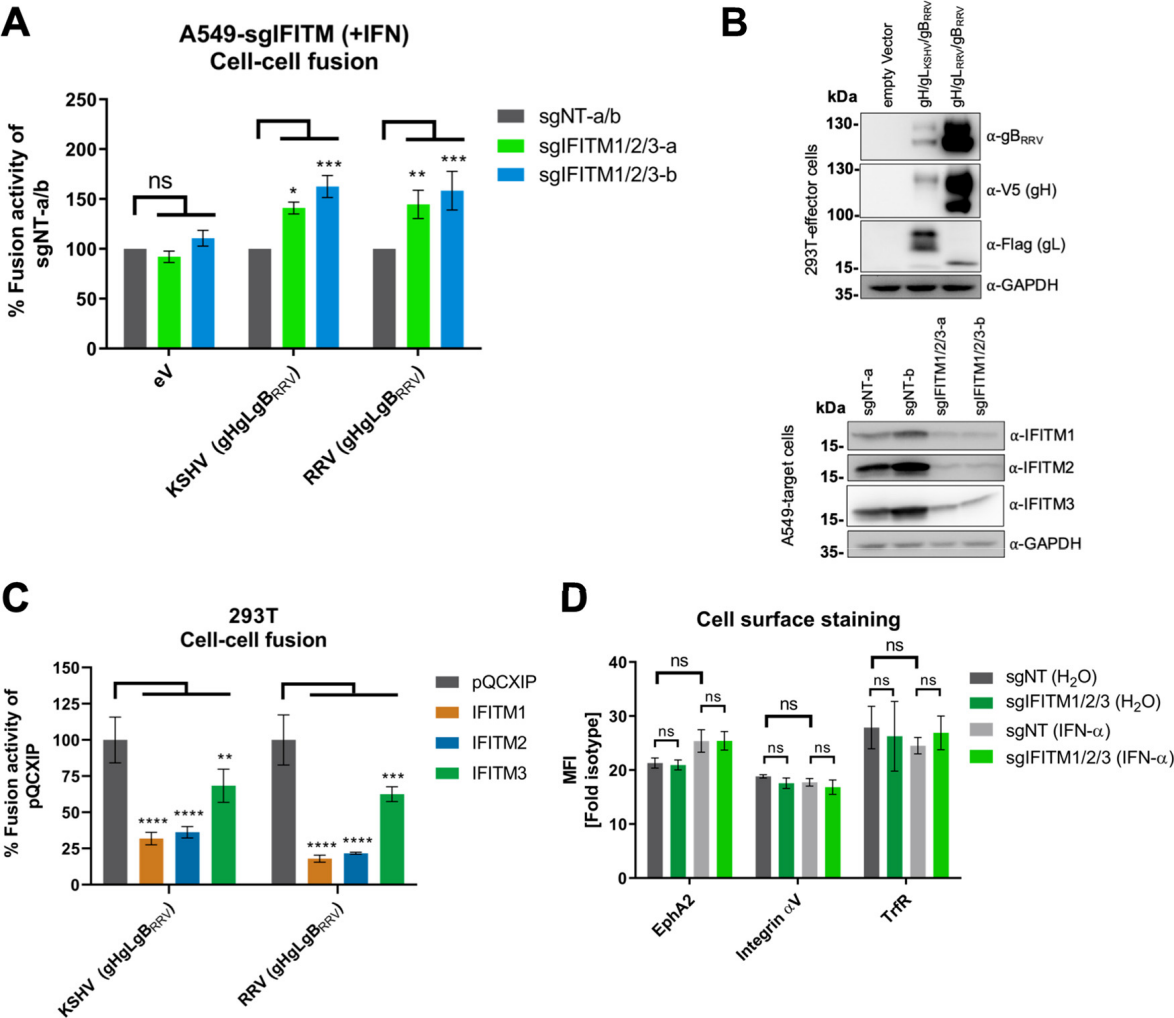
IFITMs inhibit KSHV and RRV glycoprotein-mediated cell-cell fusion. (A) Cell‐cell fusion assay. Effector cells (293T cells transfected with either empty vector [eV] or expression plasmids for the indicated viral glycoproteins together with Vp16‐Gal4 expression plasmid) were added to target cells (A549 cells transduced with a Gal4-driven TurboGFP-luciferase construct and the respective CRISPR/Cas9 sgRNA construct), which had been preincubated for 16 h with IFN-α (5,000 U/ml). After 48 h, luciferase activity was measured. Values were normalized to the mean of the two nontargeting controls, sgNT-a and sgNT-b (sgNT-a/b), which was set to 100%, for each experiment. Error bars represent standard errors of the mean of the results of four independent experiments, each performed in triplicate. Statistical significance was determined by two‐way ANOVA; *P* values were corrected for multiple comparisons by Dunnett’s method (ns, *P* > 0.05; *, *P* ≤ 0.05; **, *P* ≤ 0.01; ***, *P* ≤ 0.001; ****, *P* ≤ 0.0001). (B) The expression of proteins in 293T effector and A549 target cells after cocultivation was analyzed by Western blotting from lysates harvested for determination of luciferase activity shown in panel A using the indicated antibodies. GAPDH served as a loading control. (C) Cell‐cell fusion assay. Effector cells (293T cells transfected with expression plasmids for the indicated viral glycoproteins together with Vp16‐Gal4 expression plasmid) were added to target cells (293T cells transfected with a Gal4-driven TurboGFP-luciferase construct and the respective pQCXIP-IFITM construct). After 48 h, luciferase activity was measured. Values were averaged from three independent experiments, each performed in triplicate. The data were normalized to empty vector control pQCXIP, which was set to 100%, and error bars represent the standard deviation. Statistical significance was determined by two‐way ANOVA; *P* values were corrected for multiple comparisons by Dunnett’s method (ns, *P* > 0.05; *, *P* ≤ 0.05; **, *P* ≤ 0.01; ***, *P* ≤ 0.001; ****, *P* ≤ 0.0001). (D) A549 cells were transduced with a lentiviral vector encoding Cas9 and sgRNAs shown in Fig. 2. IFITM knockout (sgIFITM1/2/3-a, sgIFITM1/2/3-b) or control (sgNT-a and sgNT-b) cells treated with IFN-α (5,000 U/ml) or H_2_O (control) were stained for cell surface expression of the indicated proteins. The graph shows fold values for the mean fluorescence intensity over that of the isotype control averaged from two nontargeting (sgNT-a, sgNT-b) or IFITM1/2/3 knockout (sgIFITM1/2/3-a, sgIFITM1/2/3-b) transduced cells from one representative experiment performed in triplicate. Error bars represent the standard deviation. Statistical significance was determined by two‐way ANOVA; *P* values were corrected for multiple comparisons by Tukey’s method (ns, *P* > 0.05; *, *P* ≤ 0.05; **, *P* ≤ 0.01; ***, *P* ≤ 0.001; ****, *P* ≤ 0.0001).

Treatment with IFITM-targeting sgRNAs resulted in an increase of KSHV and RRV gH/gL/gB-mediated cell-cell fusion compared to nontargeting controls (Fig. 6A). Viral glycoprotein expression and IFITM1/2/3 knockout in target cells was confirmed by Western blotting (Fig. 6B). Under conditions of recombinant overexpression, all three IFITMs were capable of reducing KSHV and RRV gH/gL/gB-mediated cell-cell fusion, with IFITM1 being the most effective (Fig. 6C). To exclude the possibility that the inhibition of KSHV and RRV glycoprotein-mediated cell-cell fusion occurs in response to changes in cell surface protein composition upon IFITM1/2/3 knockout, we measured cell surface expression of a set of selected cell surface receptors. Cell surface expression of the KSHV receptors EphA2 and integrin αV as well as transferrin receptor (TrfR) remained unchanged upon IFITM1/2/3 knockout (Fig. 6D). This suggests that IFITMs reduce cell-cell fusion through a mechanism distinct from receptor regulation.

## DISCUSSION

Differences in the activity of IFITMs against several members of the herpesvirus family have already been reported (18–21, 23). Here, we report that human IFITM1 inhibits the entry of KSHV and of the closely related rhesus macaque virus RRV in a cell-specific manner. We identified inhibition of membrane fusion as a potential mechanism through which IFITMs can modulate KSHV and RRV infection.

Combined knockout of all three IFITMs enabled us to study IFITM-mediated restriction through loss of function at expression levels that are induced through IFN signaling and free from potential artifacts through overexpression-induced mislocalization. Our approach revealed that KSHV and RRV infections are enhanced upon IFITM1/2/3 knockout in A549 cells and HFF but not in HUVEC. Similarly, overexpression of individual IFITMs did not result in measurable inhibition, e.g., in SLK cells, even though they were highly effective against IAV-LP. This finding may be explained by differences in entry routes that KSHV and RRV utilize to enter these different cells. KSHV was shown to enter HFF (70) and RRV rhesus fibroblasts (33, 44) via clathrin-mediated endocytosis, whereas KSHV enters HUVEC via macropinocytosis (see Fig. S3 in the supplemental material) (42). In contrast, KSHV infects A549 cells via a pH-dependent but largely macropinocytosis-independent pathway, as indicated by sensitivity to bafilomycin A but not to EIPA (Fig. S3). While most viruses that are restricted by IFITMs enter cells via clathrin- or caveolin-mediated endocytosis, only Ebola and Marburg viruses are restricted and enter their target cells predominantly via macropinocytosis (reviewed in reference 71). However, Ebola and Marburg virus glycoproteins are activated by endosomal cathepsins, which are found mainly in endolysosomal vesicles that also contain IFITMs (reviewed in references 72 and 73). In contrast, KSHV might already fuse in acidified IFITM-negative macropinocytotic compartments and thereby avoid IFITM restriction in HUVEC, in line with the observation that even overexpression of IFITMs in HUVEC has only a minor effect on KSHV infection (Fig. S1B) and knockout has no effect at all (Fig. 3C). A question that remains unanswered is why RRV is not affected by IFITMs in HUVEC at endogenous expression levels. RRV clearly does not enter these cells through the same EIPA-sensitive pathway that KSHV uses but also does not underlie the same restriction that IAV-LP does (Fig. 3 and 4; Fig. S3). Although IFITM1/2/3 knockout enhanced KSHV and RRV infection in A549 cells and HFF, the overall contribution to the IFN-mediated block to infection was different. In HFF, the enhancement was mainly observable in IFN-α-treated cells, while in A549 cells, the enhancement was also observable in non-IFN-treated cells. In A549, the IFITM1/2/3 knockout-mediated enhancement practically cancelled out the IFN-α-mediated inhibition of KSHV and RRV infection, similar to what was observed for the highly restricted IAV-LP. Despite minor differences, IFITM1/2/3 knockout similarly impacted KSHV and RRV infection, compatible with a broadly acting mechanism like decreasing membrane fusogenicity. Vice versa, it was also shown that primate IFITMs are effective against human viruses (74, 75), in line with the high degree of conservation of IFITMs in primate species (76, 77).

Overexpression of individual IFITMs in different cells revealed IFITM1 as the major contributor to IFITM-mediated restriction of KSHV and RRV. Similar to our observations, an antiviral effect of IFITM1 in A549 cells was also identified for the alphaherpesvirus HSV-1 in IFITM1 overexpression and siRNA-mediated knockdown experiments (18), which suggests broad activity of IFITM1 against herpesviruses. Of note, an effect of IFITM1 on KSHV infection has already been described by Hussein and Akula; however, in contrast to our study, their study reported that infection by KSHV, EBV, and HSV-2 was enhanced upon overexpression of IFITM1 in the BJAB B-cell line and in HMVEC-D cells (21). While we did not observe this phenomenon in the cells analyzed in this study, our observations of cell-specific antiviral activity do not rule out the possibility that in some cells infection might actually be enhanced by IFITM1 expression. In line with this notion, overexpression of IFITM2 resulted in a mild enhancement of RRV infection in SLK cells and HFF (Fig. 4). We observed a small reduction of KSHV infection of A549 cells as well by IFITM3 overexpression, which reached significance in one set of experiments (Fig. S1A). This effect was reduced when the YxxΦ endocytic sorting motif (Δ1–21, Y20A) or the 43AS motif of IFITM3 was mutated (Fig. S1A). In contrast, RRV infection was significantly inhibited by overexpression of IFITM3 Y20A mutants and IFITM3 Y20A and Δ1–21 mutants but not wt IFITM3, in this case clearly implicating these sorting signals and by extension localization of IFITMs in their mode of action. Given the higher susceptibility of KSHV than of RRV to bafilomycin A1 (Fig. S3), we speculate that a portion of RRV particles fuses at more peripheral sites, where infection is partially restricted by IFITM1 and IFITM3 Y20A and Δ1–21 mutants. IFITM-mediated inhibition of KSHV or RRV infection was less pronounced than inhibition of IAV glycoprotein-driven entry. Several groups reported that IAV colocalized strongly with IFITM3 (15, 53, 78). We were unable to observe a pronounced colocalization of IFITMs with KSHV particles in the sense that many KSHV particles accumulate at regions of high IFITM intensity. However, we observed a clear difference in the colocalization of KSHV viral particles and IFITM1 in A549 cells and HUVEC at the 0-min time point, although the overall IFITM localization was similar in these cells (Fig. 5; Fig. S2). This observation fits well with the observation of different entry routes that KSHV utilizes to enter A549 cells and HUVEC and might explain the difference in susceptibility of KSHV toward IFITM1 between these two different cells. Interestingly, KSHV_mNeon-orf65 particles that entered the cell were frequently observed in regions with low IFITM signal (Fig. S4). While these findings argue against concentration of IFITMs at viral particles, they would be compatible with indirect mechanisms of action such as rerouting of endocytotic pathways or reduction of membrane fusogenicity.

Mechanistically, we found that IFITMs modulate KSHV and RRV glycoprotein-induced membrane fusion at IFN-α-induced levels. Overexpression of IFITM1, IFITM2, and IFITM3 revealed that all three IFITMs can in principle reduce the KSHV and RRV glycoprotein-induced cell-cell fusion to a different degree. It should be noted that overexpression of IFITMs leads to abnormal localization, thereby potentially broadening activity. This supports the theory that all IFITMs are, in principle, capable of restricting fusion (6, 16, 79), which might be counteracted by avoidance of IFITM-positive compartments. In line with our experiments, IFITM overexpression was reported to reduce the fusion activity of other viral fusion proteins, including the IAV-HA (12, 13, 15) and severe acute respiratory syndrome coronavirus 2 spike (80) proteins as well as the glycoprotein of the otherwise nonrestricted Lassa virus (15). Although cell-cell fusion does not universally mirror virus-cell fusion (81), our findings support a model of IFITM1 rendering the membrane less fusogenic. A general impact of IFITMs on membrane properties is also supported by a report that IFITMs inhibit trophoblast fusion (69). While our approach of a triple knockout was also intended to identify potential synergism between the three IFITMs, it did not do so. In HFF, IFITM1 might even counteract the mild enhancing effect that IFITM2 had on RRV infection (Fig. 4 B). Overexpression of IFITM1 was sufficient to effect inhibition with a similar magnitude as the enhancement that was observed after knockout. In light of our results and a recent report that IFITM3 blocks the IAV fusion process by increasing membrane stiffness (13), one might speculate that the three IFITMs exert their inhibitory activity through a similar mechanism at different locations.

Comparison of our experimental results in overexpression systems with those at interferon-induced expression levels and knockout of the three IFITMs allow a number of conclusions: (i) a significant discrepancy exists between results using gene knockout versus retroviral vector-driven overexpression in HUVEC; (ii) overexpression of IFITMs results in clearly aberrant morphology of HUVEC; and (iii) overexpression results in inhibition of MLV-LP, which is typically not restricted (2). These findings together with previous reports of mislocalization upon overexpression (6) and our finding that recombinant expression of the endosomal IFITM2 and IFITM3 results in inhibition of cell-cell fusion, which is strongly counterintuitive, suggest that results relying on overexpression of single IFITMs should be interpreted with caution. A recent study reported similar findings for SARS-CoV-2 (82). Gene knockout or knockdown may be essential to draw conclusions on function at naturally occurring expression levels.

Entry driven by the HA and NA glycoproteins of IAV, a respiratory pathogen, was far more potently restricted by IFITMs in fibroblasts and endothelial cells, particularly at constitutive expression levels, than in A549 lung epithelial cells. KSHV and likely RRV (83) are endotheliotropic viruses and were restricted in lung epithelial cells but not endothelial cells. This suggests that IFITMs, which are constitutively expressed at high levels in HUVEC and fibroblasts, constitute a major line of defense against disseminated infection of extrapulmonary tissues by the respiratory pathogen IAV and that KSHV and RRV may have evolved to avoid IFITM-mediated restriction in their biological niche.

## MATERIALS AND METHODS

### Cell culture.

All cell lines in this study (Table 1) were incubated at 37°C and 5% CO_2_ and cultured in Dulbecco's modified Eagle medium, high glucose, GlutaMAX, 25 mM HEPES (Thermo Fisher Scientific) supplemented with 10% fetal calf serum (FCS; Thermo Fisher Scientific) and 50 μg/ml gentamicin (PAN-Biotech) (D10) except for HUVEC, which were maintained in standard endothelial cell growth medium 2 (PromoCell), and iSLK cells (61), which were maintained in D10 supplemented with 2.5 μg/ml puromycin (InvivoGen) and 250 μg/ml G418 (Carl Roth). IFN-α treatment was performed by supplementing the respective culture medium with IFN-α 2b (Sigma; 5,000 U/ml). For seeding and subculturing of cells, the medium was removed, and the cells were washed with phosphate-buffered saline (PBS; PAN-Biotech) and detached with trypsin (PAN-Biotech). All transfections were performed using polyethylenimine (PEI; Polysciences) at a 1:3 ratio (mg DNA/mg PEI) mixed in Opti-MEM (Thermo Fisher Scientific). Cytotoxicity was measured using the CellTiter-Glo luminescent cell viability assay (Promega) according to the manufacturer’s instructions.

**TABLE 1:** Cell lines

Cell line or type^*a*^	Origin
293T cells	Kind gift from Vladan Rankovic, Göttingen, Germany, and originally purchased from the ATCC
A549 cells	Laboratory of Stefan Pöhlmann, German Primate Center–Leibniz Institute for Primate Research, Göttingen, Germany
SLK cells	RRID:CVCL_9569; NIH AIDS Research and Reference Reagent program
HFF	Laboratory of Klaus Korn, Universitätsklinikum Erlangen, Institute for Clinical and Molecular Virology, Erlangen, Germany
RF	Laboratory of Rüdiger Behr, German Primate Center–Leibniz Institute for Primate Research, Göttingen, Germany
HUVEC	PromoCell
iSLK cells	Kind gift from Don Ganem (61)

a HFF, human foreskin fibroblasts; RF, rhesus monkey fibroblasts; HUVEC, human vascular endothelial cells.

### Retroviral vectors and pseudotyped lentiviral particles.

Retroviruses, lentiviruses, and lentiviral pseudotypes were produced by PEI-mediated transfection of 293T cells (plasmids are listed in Table 2). For retrovirus production, plasmids encoding gag/pol, pMD2.G encoding VSV-G, and the respective pQCXIP contructs were transfected (ratio, 1.6:1:1.6). For production of lentiviruses used for transduction, psPAX2 encoding gag/pol, pMD2.G encoding VSV-G, and the respective lentiviral construct, Gal4-driven TurboGFP-luciferase reporter lentivirus (AX526) or plentiCRISPRv2, were used (ratio, 2.57:1:3.57). For lentiviral pseudotypes psPAX2, pLenti CMV GFP Neo and expression plasmids for pCAGGS IAV_WSN-HA and pCAGGS IAV_WSN-NA for IAV-LP or paMLV_env for MLV-LP were used (ratio, 1:1.4:2.4). Viruses were harvested twice, 24 to 48 h and 72 to 96 h after transfection, passed through a 0.45-μm CA filter, and frozen at −80°C. Transduction was performed by adding retroviruses and lentiviruses to cells for 48 h. Afterwards, selection was performed using 10 μg/ml puromycin (InvivoGen; pQCXIP and plentiCRISPRv2 constructs) or 10 μg/ml blasticidin (InvivoGen; AX526 lentivirus).

**TABLE 2:** Plasmids

Plasmid	Source	Reference/identifier
psPAX2	Addgene (kind gift from Didier Trono)	Addgene no. 12260
VSV-G (pMD2.G)	Addgene (kind gift from Didier Trono)	Addgene no. 12259
plentiCRISPRv2	Addgene (kind gift from Feng Zhang)	Addgene no. 52961 (57)
gag/pol	Addgene (kind gift from Tannishtha Reya)	Addgene no. 14887
pLenti CMV GFP Neo	Addgene (kind gift from Eric Campeau and Paul Kaufman)	Addgene no. 17447
AX526 (Gal4-driven TurboGFP-luciferase reporter lentivirus)	Laboratory of Alexander Hahn	38
Gal4-TurboGFP-Luc (Gal4-driven TurboGFP-luciferase reporter plasmid)	Laboratory of Alexander Hahn	81
Vp16-Gal4	Laboratory of Alexander Hahn	81
pCAGGS IAV_WSN-HA	Laboratory of Michael Farzan	3
pCAGGS IAV_WSN-NA	Laboratory of Stefan Pöhlmann	3
paMLV_env	Laboratory of Michael Farzan	3
pQXCIP	Laboratory of Stefan Pöhlmann	3
pQCXIP-IFITM1	Laboratory of Stefan Pöhlmann	3
pQCXIP-IFITM2	Laboratory of Stefan Pöhlmann	3
pQCXIP-IFITM3	Laboratory of Stefan Pöhlmann	3
pQCXIP-IFITM3-Δ1-21	Laboratory of Stefan Pöhlmann	
pQCXIP-IFITM3-Y20A	Laboratory of Stefan Pöhlmann	
pQCXIP-IFITM3-43AS	Laboratory of Stefan Pöhlmann	
pEPkan-S	Addgene (kind gift from Nikolaus Osterrieder)	Addgene no. 41017

### Production of KSHV, KSHV_mNeon-orf65, and RRV.

For the construction of KSHV_mNeon-orf65, the GFP open reading frame of BAC16 was replaced with a Zeocin resistance gene by amplifying the resistance gene from pcDNA6 (Invitrogen) using Phusion PCR (NEB) and primers BAC16_downstream_of_GFP_STOP_overhang_plus_Zeo_3′ and BAC16_upstream_of_GFP_ATG_antisense_strand_overhang_plus_EM7_P_start and inserting it into BAC16 via recombination. A shuttle construct, Ax185_ pCNSmNeonGreen_Kana, was created by inserting the i-SceI/Kanamycin cassette of pEPkan-S (84) into pNCSmNeonGreen using primers mNeonGreen_463-482_for plus mNeonGreen_504-523_rev for the vector and EPKansS_reverse_mNeon_463-482_ov plus EPKans_forward_mNeon_504-523_ov for the insert, followed by Gibson assembly. KSHV_mNeon-orf65 was generated by inserting the mNeonGreen cassette 5′ of the first amino acid of orf65 with the addition of a glycine-serine linker according to the protocol described by Tischer et al. (84). The recombination cassette was generated using primers mNeon-GS-KSHVorf65_for plus mNeon-GS-KSHVorf65_rev and Ax185_ pCNSmNeonGreen_Kana as a template.

Infectious KSHV and RRV reporter viruses were produced as described previously using the iSLK cell system for KSHV and primary rhesus monkey fibroblasts for RRV (36). See Table 3 for oligonucleotide sequences.

**TABLE 3:** Oligonucleotides

Oligonucleotide	Sequence
BAC16_downstream_of_GFP_STOP_overhang_plus_Zeo_3’	GGCGGAATTCCTCTAGTGCGGCCGAGTCGCGGCCGCTTTATCAGTCCTGCTCCTCGGCC
BAC16_upstream_of_GFP_ATG_antisense_strand_overhang_plus_EM7_P_start	GTAAGCTTGGTACCGAGCTCGGATCCACTAGTCCGCCACCTGTTGACAATTAATCATCGG)
mNeonGreen_463-482_for	TACCCCAACGACAAAACCAT
mNeonGreen_504-523_rev	TGCCATTTCCAGTGGTGTAA
EPKansS_reverse_mNeon_463-482_ov	ATGGTTTTGTCGTTGGGGTACAACCAATTAACCAATTCTGATTAG
EPKans_forward_mNeon_504-523_ov	TTACACCACTGGAAATGGCAGGATGACGACGATAAGTAGGGATAAC
mNeon-GS-KSHVorf65_for	TGTTGCGGGAAGTGTTCCTCCTGAGGCTATTTCGCCCGCCTGTGTGGAAGATGGTGAGCAAGGGC
mNeon-GS-KSHVorf65_rev	TGATCCAGTCGCTCCTGGATCACGGGGTCTCTCACCTTAAAGTTGGACATGCTTCCCTTGTACAGCTCGTCC

### Western blotting.

Western blotting was performed as described previously (36) using the respective antibodies (Table 4).

**TABLE 4:** Antibodies

Assay type and/or target	Antibody				Secondary antibody			
	Manufacturer	Clone/catalog no.^*a*^	Species	Dilution	Type	Manufacturer	Species	Dilution
Western blotting								
IFITM1	R&D Systems	AF4827	Goat	1:500	Anti-goat HRP-coupled	Proteintech	Rabbit	1:5,000
IFITM2	Proteintech	66137-1-lg	Mouse	1:500–1:1,000	Anti-mouse HRP-coupled	Dianova	Donkey	1:1,000
IFITM3	Cell Signaling Technology	D8E8G	Rabbit	1:1,000	Anti-rabbit HRP- coupled	Life Technologies	Goat	1:1,000
c-Myc epitope	Santa Cruz Biotechnology	9E10	Mouse	1:1,000	Anti-mouse HRP-coupled	Dianova	Donkey	1:1,000
MxA	R&D Systems	AF7946	Goat	1:1,000	Anti-goat HRP-coupled	Proteintech	Rabbit	1:5,000
GAPDH	GenScript	NA	Mouse	1:15,000	Anti-mouse HRP-coupled	Dianova	Donkey	1:1,000
V5 tag	Bio-Rad	NA	Mouse	1:1,000	Anti-mouse HRP-coupled	Dianova	Donkey	1:1,000
DYKDDDDK (Flag) tag	Cell Signaling Technology	D6W5B	Rabbit	1:1,000	Anti-rabbit HRP- coupled	Life Technologies	Goat	1:1,000
RRV gB	Scott W. Wong (Oregon Health & Science University)	3H8.1	Mouse	1:1,000	Anti-mouse HRP-coupled	Dianova	Donkey	1:1,000
Flow cytometry								
IgG1 Isotype	Thermo Fisher Scientific	NA	Mouse	1:500	Anti-mouse Alexa Fluor 647	Life Technologies	Donkey	1:500
EphA2	Merck	Clone F2-27	Mouse	1:500	Anti-mouse Alexa Fluor 647	Life Technologies	Donkey	1:500
Integrin alpha V/CD51	R&D Systems	P2W7	Mouse	1:500	Anti-mouse Alexa Fluor 647	Life Technologies	Donkey	1:500
CD71 (TrfR)	Thermo Fisher Scientific	OKT9	Mouse	1:500	Anti-mouse Alexa Fluor 647	Life Technologies	Donkey	1:500
Immunofluorescence								
IFITM1	R&D Systems	AF4827	Goat	1:250	Anti-goat Alexa Fluor 594 or anti-goat Alexa Fluor 647	Life Technologies	Donkey	1:500
IFITM2	Proteintech	12769-1-AP	Rabbit	1:250	Anti-rabbit Alexa Fluor 594 or anti-rabbit Alexa Fluor 647	Life Technologies	Donkey	1:500
IFITM3	Cell Signaling Technology	D8E8G	Rabbit	1:250	Anti-rabbit Alexa Fluor 594 or anti-rabbit Alexa Fluor 647	Life Technologies	Donkey	1:500
								
EEA1	BD Laboratories	610456	Mouse	1:400	Anti-mouse Alexa Fluor 647	Life Technologies	Donkey	1:500
LAMP-1	Santa Cruz Biotechnology	H5G11	Mouse	1:750	Anti-mouse Alexa Fluor 647	Life Technologies	Donkey	1:500
								
Directly labeled probes					Phalloidin-iFluor 647 conjugate	AAT Bioquest		1:1,000

a NA, not applicable.

### CRISPR/Cas9-mediated knockout of immune-related IFITMs.

IFITM1, IFITM2, and IFITM3 knockout cell pools were generated by CRISPR/Cas9-mediated knockout by following the protocol described by Sanjana et al. (57), except that PEI transfection was used. In short, the cells intended for knockout were transduced with lentiviruses harboring the CRISPR/Cas9 gene and sgRNAs targeting IFITM1-3 (sgIFITM1/2/3-a, sgIFITM1/2/3-b) or nontargeting sgRNAs (sgNT-a, sgNT-b). For detection of CRISPR/Cas9-mediated knockout, the cells were treated with IFN-α (5,000 U/ml) for 16 h. Thereafter, the cells were harvested and subjected to Western blot analysis.

### Infection experiments.

IFITM-overexpressing cells were seeded in 48-well or 96-well plates at 90% confluence 16 h prior to infection. IFITM1/2/3 knockout cells were seeded in 48-well plates at 70% to 80% confluence. After attachment, cells were treated with IFN-α (5,000 U/ml) or H_2_O (control) for 16 h prior to infection with either KSHV, RRV, IAV-LP, or MLV-LP. For endocytosis inhibitor treatment, the cells were seeded into 96-well plates at 90% confluence and were incubated for 30 min at twice the indicated concentration before virus was added. For EIPA treatment, the medium was changed after 12 h due to toxicity. At 48 h postinfection, cells were trypsinized, trypsin activity was inhibited by adding 5% FCS in PBS, and the cells were washed and fixed with a final concentration of 4% methanol-free formaldehyde (Roth) in PBS. Infection was determined by detection of GFP^+^/YFP^+^ cells using an LSRII flow cytometer or ID7000 (Sony); at least 5,000 cells were analyzed.

### Cell-cell fusion assay.

293T effector cells were seeded in 6-well plates or 10-cm dishes at 70% to 80% confluence and transfected with either empty vector, gH/gL_KSHV_ gB_RRV_, or gH/gL_RRV_gB_RRV_ and Vp16-Gal4 expression plasmids. 293T cells transfected with Gal4-TurboGFP-Luc and pQCXIP-IFITM1-3 were seeded in 48-well plates at 50,000 cells/well. A549 cells double transduced with lentiviruses encoding a Gal4-driven TurboGFP-luciferase reporter and lentiviruses encoding the CRISPR/Cas9 gene and the respective sgRNAs were seeded in 96-well plates at 20,000 cells/well; 6 h after seeding, the cells were treated with IFN-α (5,000 U/ml) for 16 h. Cell-cell fusion was started by adding the glycoprotein-expressing effector cells to the target cells in a 1:1 ratio. After 48 h, the cells were lysed in luciferase cell culture lysis reagent (Promega) and luciferase activity was determined using the Beetle-Juice luciferase assay (PJK Biotech) according to the manufacturer’s instructions and a BioTek Synergy 2 plate reader.

### Flow cytometry.

For detection of cell surface proteins, A549 IFITM1/2/3 knockout cells were H_2_O or IFN-α treated for 16 h, washed with PBS, detached using EDTA/EGTA (5 mM/5 mM) at 37°C, and washed with cold PBS (4°C). The cells were fixed with 4% methanol-free formaldehyde for 5 min and washed twice with PBS. Following blocking with 10% FCS (blocking buffer) in PBS, the cells were incubated with primary antibody (Table 4) in blocking buffer for 90 min at 4°C. After washing with PBS, the cells were incubated with secondary antibody (Table 4) in blocking buffer for 45 min at room temperature (RT) in the dark. The cells were washed and postfixed with 2% methanol-free formaldehyde in PBS. Analysis was performed using an LSRII flow cytometer (BD Biosciences) and Flowing software (University of Turku, version 2.5).

### Immunofluorescence.

A549 cells or HUVEC were seeded on 12-mm coverslips (YX03.1; Carl Roth) in 24-well plates at 150,000 cells/well. After attachment, the cells were treated with either H_2_O (control) or IFN-α (5,000 U/ml) for 16 h and cold KSHV_mNEON-ORF65 was added. Cells were centrifuged (4,200 rpm, 4°C, 30 min), followed by a 10-min incubation at 4°C. After 3 washes with cold PBS, cells were either fixed in 4% methanol-free formaldehyde in PBS for 10 min (0-min time point) or shifted to 37°C after addition of D10 (A549 cells) or endothelial cell growth medium 2 (HUVEC). At the indicated time points (Fig. 5; Fig. S4), cells were washed once in PBS and fixed in 4% methanol-free formaldehyde in PBS for 10 min. After fixation, cells were washed three times in PBS. For Fig. 5A, uninfected cells were directly fixed after 16 h of IFN-α treatment as described above. Cell permeabilization and blocking were performed in immunofluorescence (IF) buffer (5% FCS, 0.05% saponin [Sigma] in PBS) for 1 h. Primary antibody (Table 4) incubation was performed in IF buffer overnight at 4°C or 2 h at RT. Secondary antibody (Table 4) incubation or incubation with a directly labeled phalloidin probe was performed after three washes with IF buffer for 1 h at RT. Cells were washed once in IF buffer and stained with Hoechst 33342 at 1:10,000 in PBS (catalog no. 62249; Thermo Scientific) for 5 min, followed by a final wash with PBS. The coverslips were dried and mounted in anti-fade fluorescence mounting medium (ab104135; abcam). Images were acquired on a confocal laser scanning microscope (Zeiss LSM800). Laser intensity and signal amplification were maintained for each experiment between different conditions for each antibody staining. All images were processed using Fiji/ImageJ software. For the quantification of colocalization of mNEON and IFITM signals, the Fiji “colocalization” plugin was used. The automatic threshold for IFITM signals was determined using the “IsoData” method, averaged for all analyzed images per experiment, and used in the colocalization analysis of all respective IFITM stainings in this experiment. The threshold for mNEON signals was kept constant in all analyses. Quantification of colocalized particles was performed on the “colocalizated points 8-bit” output image using the Fiji built-in “find maxima” function. Quantification of total mNEON-positive particles was performed using the Fiji built-in “find maxima” function on the mNEON channel after thresholding identical to the colocalization analysis. For each experiment and time point, at least three images were analyzed and averaged. For representative images, automatic contrast enhancement on all channels was performed. All images were smoothed prior to processing.
