# Supplementary figures and images for: Interferon-Induced Transmembrane Proteins Inhibit Infection by the Kaposi’s Sarcoma-Associated Herpesvirus and the Related Rhesus Monkey Rhadinovirus in a Cell-Specific Manner
Source: mBio. 2021 Dec 21;12(6):e02113-21. doi: 10.1128/mBio.02113-21 (PMC8689460; doi:10.1128/mBio.02113-21)

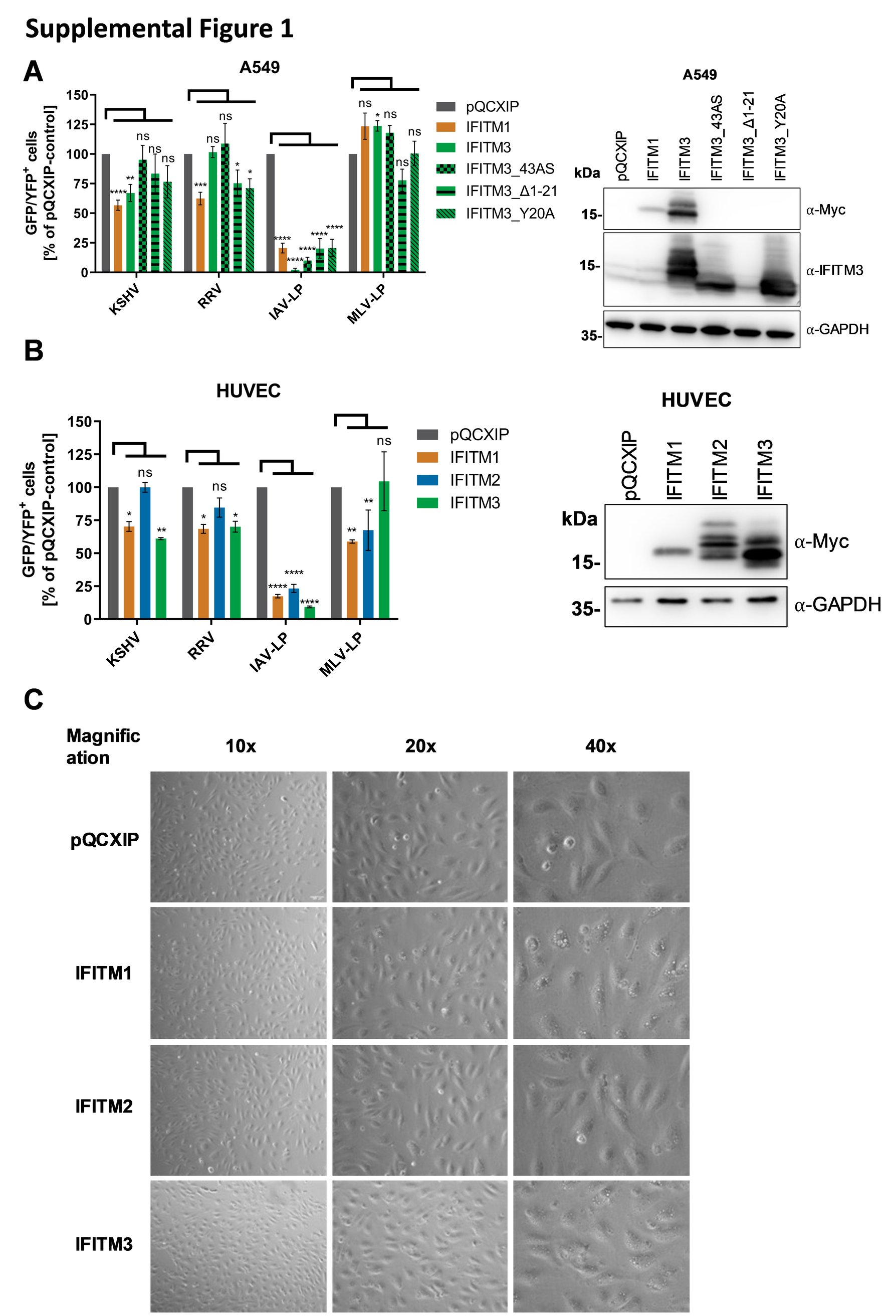

Supplement: FIG S1 [file mbio.02113-21-sf001.tif]

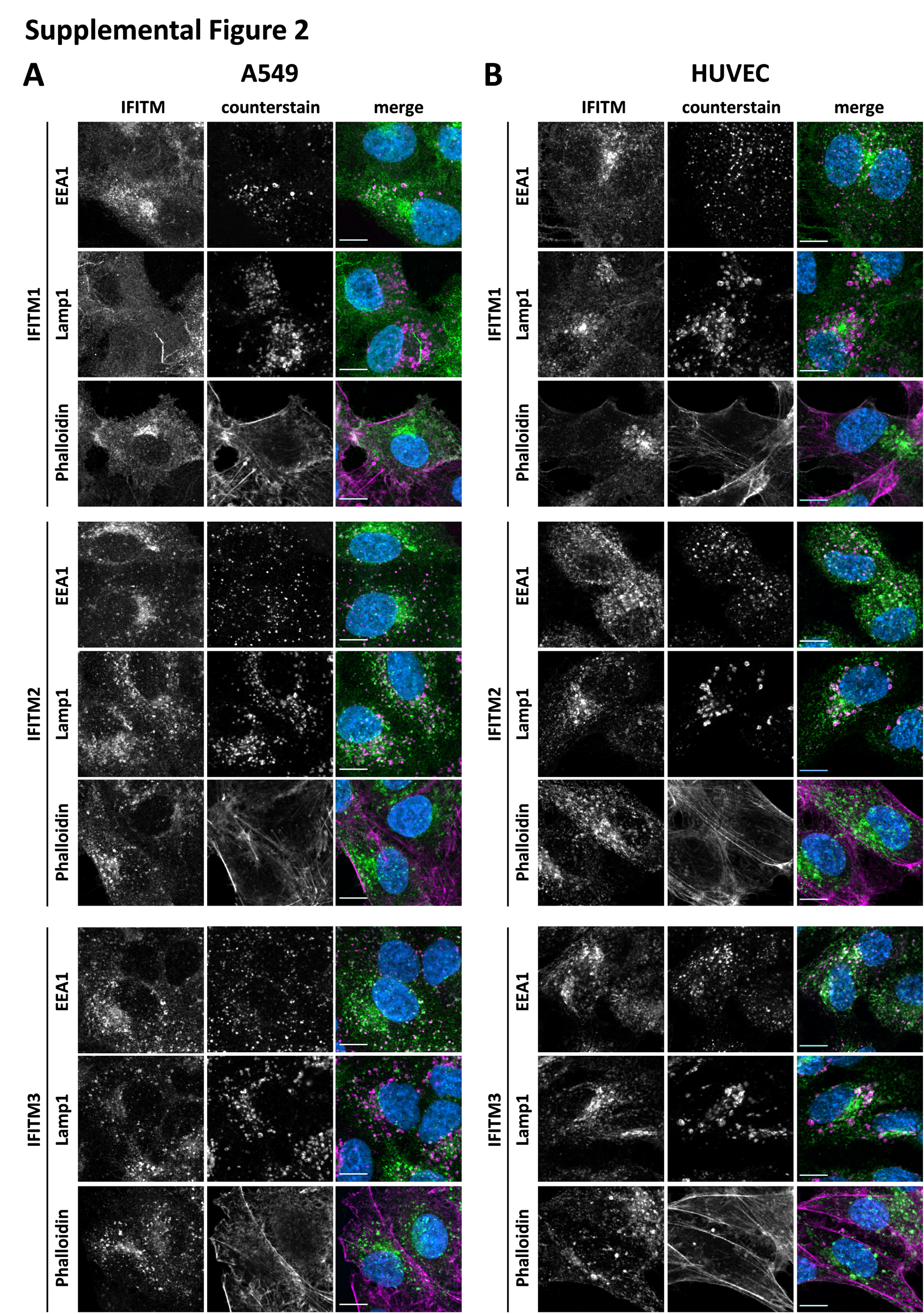

Supplement: FIG S2 [file mbio.02113-21-sf002.jpg]

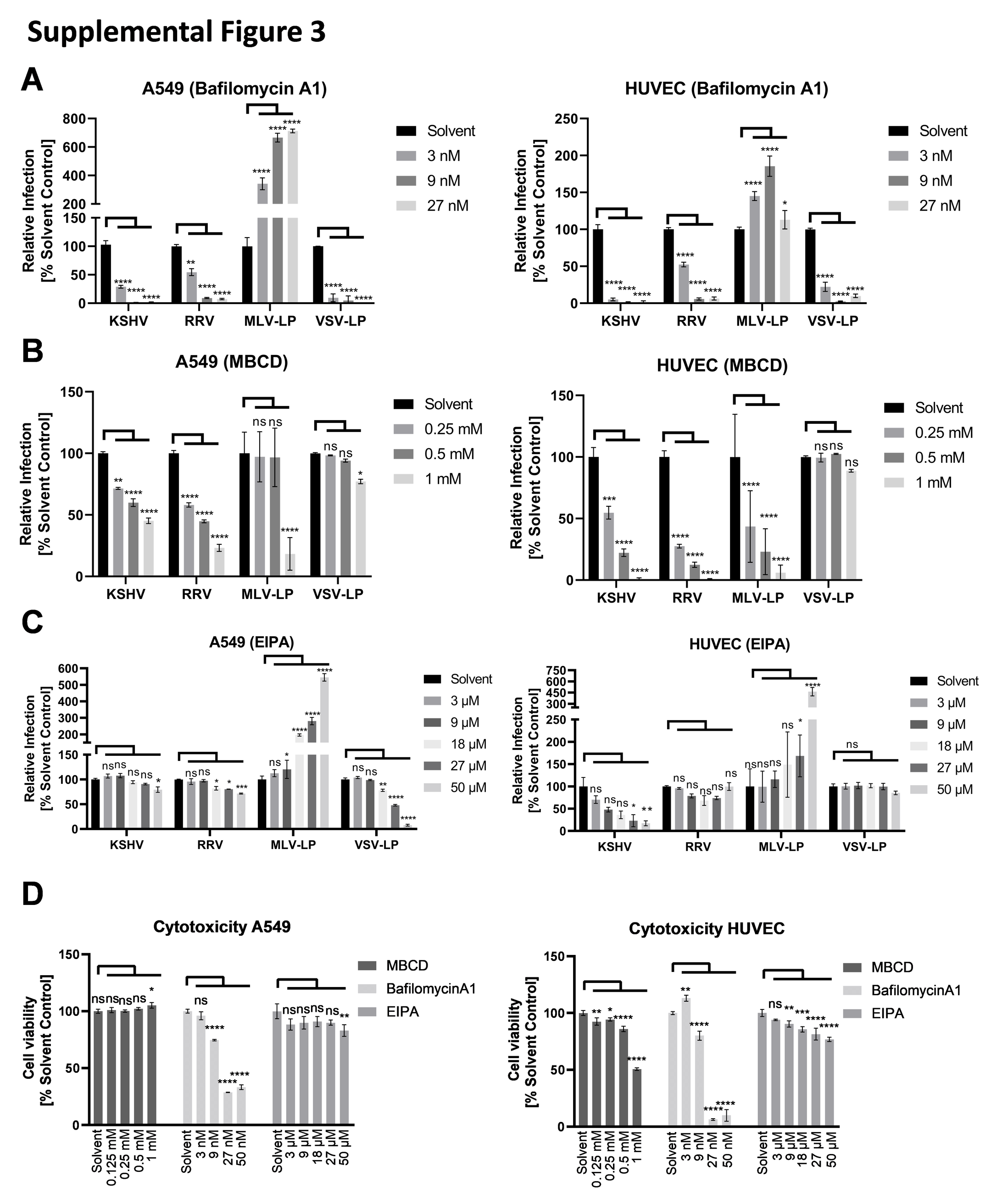

Supplement: FIG S3 [file mbio.02113-21-sf003.tif]

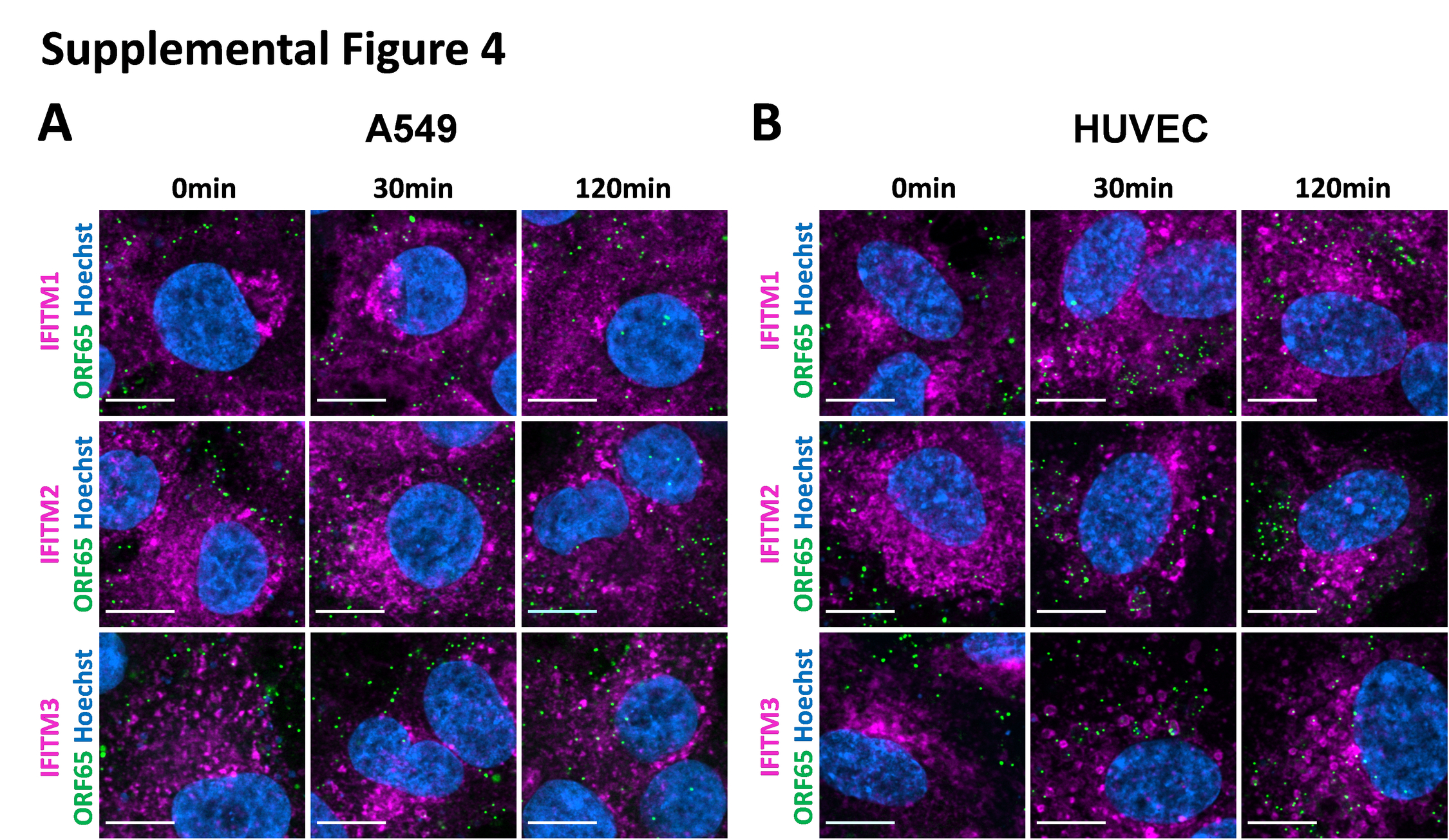

Supplement: FIG S4 [file mbio.02113-21-sf004.jpg]

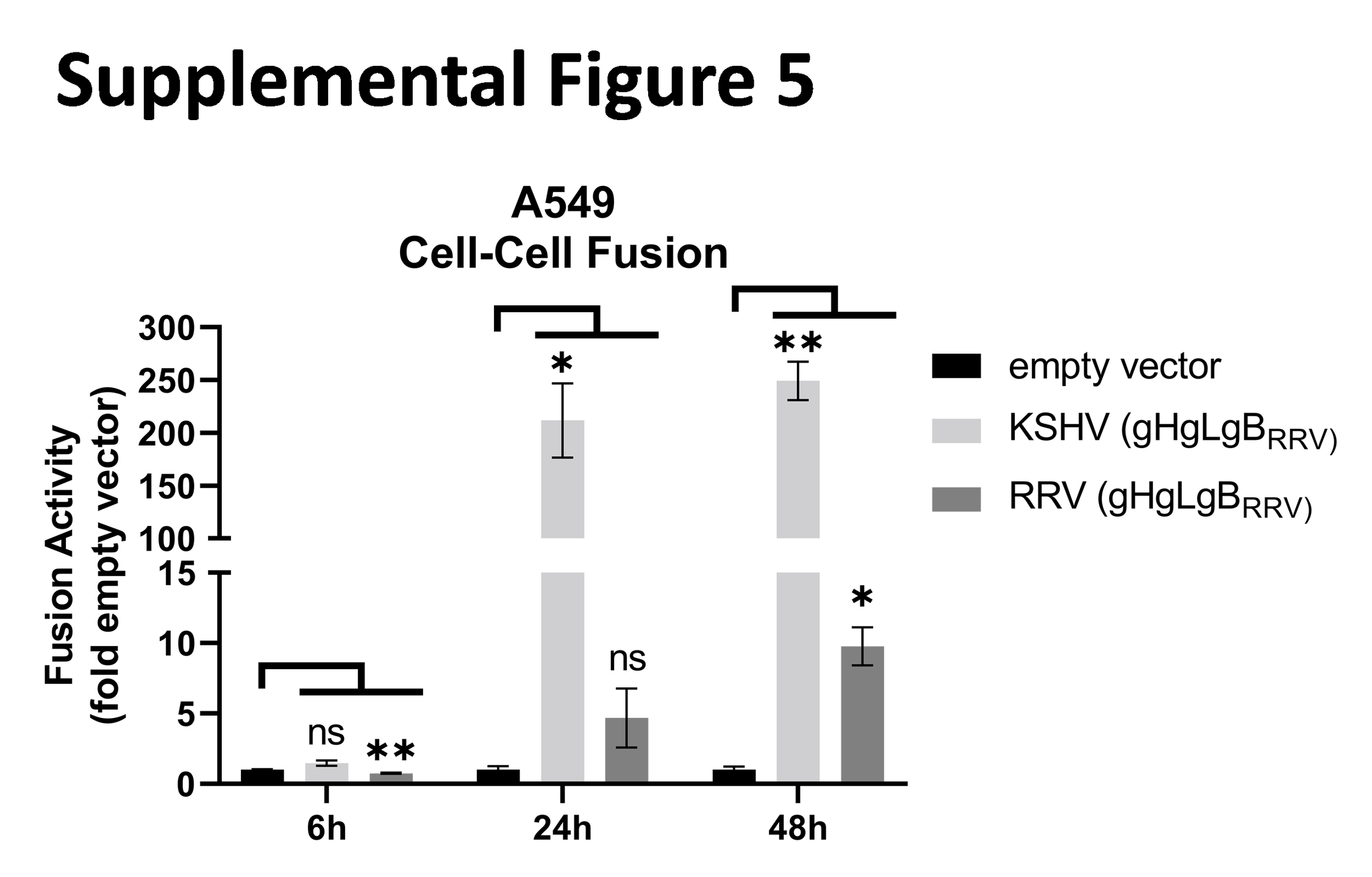

Supplement: FIG S5 [file mbio.02113-21-sf005.tif]
